# La-related protein 6 controls ciliated cell differentiation

**DOI:** 10.1186/s13630-017-0047-7

**Published:** 2017-03-23

**Authors:** Zarko Manojlovic, Ryan Earwood, Akiko Kato, Diana Perez, Oscar A. Cabrera, Ruth Didier, Timothy L. Megraw, Branko Stefanovic, Yoichi Kato

**Affiliations:** 1 Department of Biomedical Sciences, Florida State University College of Medicine, 1115W. Call Street, Tallahassee, FL 32306-4300 USA; 2 Department of Translational Genomics, Keck School of Medicine of University of Southern California, Los Angeles, CA 90089-9601 USA

**Keywords:** LARP6, Cilia, mcidas, *Xenopus*

## Abstract

**Background:**

La-related protein 6 (LARP6) is an evolutionally conserved RNA-binding protein. Vertebrate LARP6 binds the 5′ stem-loop found in mRNAs encoding type I collagen to regulate their translation, but other target mRNAs and additional functions for LARP6 are unknown. The aim of this study was to elucidate an additional function of LARP6 and to evaluate the importance of its function during development.

**Methods:**

To uncover the role of LARP6 in development, we utilized Morpholino Oligos to deplete LARP6 protein in *Xenopus* embryos. Then, embryonic phenotypes and ciliary structures of LAPR6 morphants were examined. To identify the molecular mechanism underlying ciliogenesis regulated by LARP6, we tested the expression level of cilia-related genes, which play important roles in ciliogenesis, by RT-PCR or whole mount in situ hybridization (WISH).

**Results:**

We knocked down LARP6 in *Xenopus* embryos and found neural tube closure defects. LARP6 mutant, which compromises the collagen synthesis, could rescue these defects. Neural tube closure defects are coincident with lack of cilia, antenna-like cellular organelles with motility- or sensory-related functions, in the neural tube. The absence of cilia at the epidermis was also observed in LARP6 morphants, and this defect was due to the absence of basal bodies which are formed from centrioles and required for ciliary assembly. In the process of multi-ciliated cell (MCC) differentiation, mcidas, which activates the transcription of genes required for centriole formation during ciliogenesis, could partially restore MCCs in LARP6 morphants. In addition, LARP6 likely controls the expression of *mcidas* in a Notch-independent manner.

**Conclusions:**

La-related protein 6 is involved in ciliated cell differentiation during development by controlling the expression of cilia-related genes including *mcidas*. This LARP6 function involves a mechanism that is distinct from its established role in binding to collagen mRNAs and regulating their translation.

**Electronic supplementary material:**

The online version of this article (doi:10.1186/s13630-017-0047-7) contains supplementary material, which is available to authorized users.

## Background

The cilium is a motile or non-motile cellular protrusion that is essential for cell physiology, development and organ homeostasis [1–3]. The ciliary motile function generates fluid flow in a directional manner in multicellular organisms, and also serves to propel cells such as protists and spermatozoa. Non-motile primary cilia sense physical and biochemical extracellular signals in a variety of contexts. Ciliary defects are associated with a wide spectrum of severe human diseases, called ciliopathies [4].

Recent progress in RNA biology has shown that RNA-binding proteins (RBPs) are important post-transcriptional regulators [5]. However, the only known RBP linked to ciliogenesis thus far is Bicaudal C (BicC) [6, 7]. BicC mediates the alignment of cilia at the left–right organizer, possibly by regulating the activity of the canonical Wnt pathway through Dvl2 and/or RNA silencing in P-bodies [6]. Although the post-transcriptional regulation by RBPs is likely an important level of regulatory control for ciliogenesis, the RBPs involved and their regulatory mechanisms remain largely undiscovered.

LARP6 is a member of the LARP family of RBPs which carries a La motif (LAM) and a RNA recognition motif (RRM) [8–10]. LARP family member proteins have been implicated in regulating transcription and/or mRNA translation as well as maturation of tRNAs. The RNA-binding properties of LARP proteins are determined by subtle sequence variations within their LAM and RRM domains. LARP6 was originally reported to be involved in the programmed cell death of intersegmental muscle in the moth *M. sexta* [11]. Recently, vertebrate collagen mRNAs including alpha1 (I), alpha2 (I) and alpha1 (III) have been identified as binding targets for LARP6 [12]. LARP6 regulates the generation of heterotrimeric type I collagen molecules by interacting with the 5′ stem-loop structure in the 5′ UTR of these collagen mRNAs and affecting their translation. The binding to the 5′ stem-loop requires the LAM–RRM domain (La-module) of LARP6 and is strictly sequence specific [12]. LARP6 also associates with non-muscle myosin filaments, vimentin intermediate filaments, RNA helicase A, serine–threonine kinase receptor-associated protein (STRAP) and FK506 binding protein 3 (FKBP3) [13–17]. These interactions regulate coordinated translation of collagen mRNAs to achieve 2:1 ratio of the type I collagen subunits.

A role of LARP6 in myogenesis has also been described. LARP6 stimulated the differentiation and self-renewal of mouse C_2_C_12_ cells during in vitro myogenesis and the formation of myotubes in zebrafish embryos [18]. These functions of LARP6 are probably unrelated to binding collagen mRNAs but it is unclear if a role of LARP6 in myogenesis is through the translational regulation of some other target mRNAs. Since target mRNAs other than type I collagen mRNAs have not been identified to date, the principle role of LARP6 is currently thought to be the regulation of collagen synthesis in terminally differentiated fibroblasts and myofibroblasts.

Here, we report that LARP6 is required for the formation of cilia in *Xenopus* embryos. Knockdown of LARP6 in *Xenopus* embryos resulted in neural tube closure defects coincident with loss of cilia, both primary cilia and floor plate cilia, in the neural tube. Furthermore, MCCs at the epidermis were also absent in LARP6 morphants. Our results demonstrate that the loss of the transcription regulator *mcidas*, a critical factor in centriole assembly and ciliogenesis in MCCs, is in part responsible for the lack of ciliary basal bodies formation in the epidermis of LARP6 morphants. However, additional factors whose expression is controlled by LARP6 appear to also be involved in this process. These data show that LARP6 is a regulatory factor which is required for the differentiation of ciliated cells.

## Methods

### Embryo manipulations

Eggs were artificially fertilized using testis homogenates and cultivated in 0.1 × Marc’s Modified Ringer’s solution (MMR) [19]. Embryos were staged according to Nieuwkoop and Faber [20].

### DNA constructs

*Xenopus laevis* LARP6 (accession number DR716536) clone for whole mount in situ hybridization (WISH) probes and microinjection were purchased from Thermo Scientific. To generate WISH probes, LARP6-*Eco*RI/*Not*I fragments were sub-cloned into pBluescript KS vector. To generate microinjection RNA, *X. laevis* LARP6 was isolated by PCR with following primers and was sub-cloned into pCS2+-2HA vector (pCS2+xlL6-2HA). LARP6: forward 5′-GCGGATCCATTCCTGCCTGATAGAAGCT-3′, reverse 5′-GCGTCGACATAAACACTGAACGGGCTTA-3′. pExpress1 *X. laevis* mcidas (accession number BC124892) was used for the synthesis of mcidas mRNA. Other plasmids for WISH probes including sox2 and mcidas or for microinjection including human LARP6 (HA-hL6), Δ1-300, Δ1-478, Δ135Δ264 and LARP6-GFP (L6-GFP) were described in previous publication [12, 15, 21, 22].

### Microinjection of synthetic mRNA and Morpholino Oligos

Capped synthetic mRNAs were generated by in vitro transcription with SP6 polymerase, using the mMessage mMachine kit (Ambion/Life Technologies). For microinjections, embryos were injected with 5–10 nl of the specified amount of mRNA in 3% Ficoll in 0.1 × MMR and cultured in 0.1 × MMR until the desired stage. The site of injection was determined based on the cell fate map of *Xenopus* embryos [23]. nucß-gal mRNA for WISH and membrane RFP (memRFP) mRNA as well as membrane GFP (memGFP) mRNA for immunohistochemistry were injected as a lineage tracer. Antisense Morpholino Oligos (MO) and standard control MO were purchased from Gene Tools. Sequences of MO used in this work are followed. Control MO (conMO): CCTCTTACCTCAGTTACAATTTATA, LARP6 MO (L6MO): TCTCCTCGGGCTCCTCCATGTCACT. Mismatches between LARP6 S, whose sequence was used in this study, and LARP6 L (accession number BJ030119) are four nucleotides including missing three nucleotides in the middle of MO.

### Semi-quantitative RT-PCR analysis

Total RNA was isolated with TRIzol reagent (Life Technologies) according to the manufacturer’s instructions. Semi-quantitative RT-PCR was performed as described previously [15, 24]. Sequences of primers used; LARP6: forward 5′-TGTGCGCAAAAACAAGTCTC-3′, reverse 5′-TTGTTCACAAGGGCTACTCC-3′; mcidas: forward 5′-TAGAGGGCGCACAATTACCT-3′, reverse 5′-GCCACCAGTGGTTTTAATGG-3; mab21-l3: forward 5′-ATGCCCTGGCTGATAAGTTG-3′, reverse 5′-TAGAATTGTAGGCGGGTTGG-3; gmnc: forward 5′-CTCCAAACCTTGGGACTGAA-3′, reverse 5′-TCACTTTCTGGCAGTGATGC-3. Other primers were described in previous publication [25, 26].

### Beta-galactosidase staining and whole mount in situ hybridization

Embryos were fixed with MEMFA (0.1 M MOPS, 2 mM EGTA [pH8.0], 1 mM MgSO_4_ and 3.7% formaldehyde) containing 0.02% Triton-X for 30 min at room temperature. Galactosidase activity was visualized with the RedGal substrate (Research Organics) in staining buffer (5 mM K_3_[Fe(CN)_6_], 5 mM K_4_[Fe(CN)_6_], 2 mM MgCl_2_ in PBS). After staining, embryos were re-fixed with MEMFA for 30 min. WISH was performed as described previously [27–29] using BM purple (Roche Applied Science) for the chromogenic reaction. RNA probes for WISH were labeled with Digoxigenin (Roche Applied Science).

### Whole mount fluorescence immunohistochemistry

Published procedures were used for staining [30, 31] and thick sectioning for the neural tube [32] with minor modifications. Embryos were fixed in MEMFA for either 2.5 h at room temperature or overnight at 4 °C for anti-acetylated-α-tubulin, anti-B9D1 and anti-γ-tubulin antibodies or in methanol for 48 h at 4 °C for anti-centrin antibody. Fixed embryos were dehydrated completely in methanol at −20 °C for at least several hours and rehydrated consecutively with PBS. After rinsing in PBT (0.1% Triton X-100 in PBS), embryos were incubated with 10% goat serum in PBT at room temperature for at least 1 h. Samples were incubated with mouse anti-acetylated-α-tubulin antibody (1:500, Sigma), rabbit anti-B9D1 antibody (1:50, abnova), rabbit anti-γ-tubulin antibody (1:50, abcam) and mouse anti-centrin antibody (1:50, Millipore) overnight at 4 °C. Primary antibodies were recognized with Cy2 donkey anti-mouse IgG antibody (1:500, Jackson ImmunoResearch) and Alexa Flour 648 goat anti-rabbit IgG antibody (1:500, Life Technologies), respectively. Antibodies were diluted in 10% goat serum in PBT. Images were taken by confocal microscopy.

### Immunoblotting

*Xenopus* embryos were homogenized in lysis buffer [20 mM Tris–HCl: pH 8.0, 5 mM MgCl_2_, 1 mM EDTA, 50 mM KCl, 10% glycerol, 0.5% Triton-X, Protease Inhibitor Cocktail (Roche Applied Science)], and embryonic protein extracts were used for immunoblotting with rabbit anti-γ-tubulin (1:1000, abcam), mouse anti-centrin (1:1000, Millipore) and rabbit anti-β-actin (1:2000, Thermo Scientific) antibodies.

### Statistical methods

Statistical analyses were carried out using Student’s *t* test to calculate *P* values. A *P* value <0.05 was considered to be significant.

## Results

### LARP6 morphants have neural tube closure defects

**Fig. 1:**
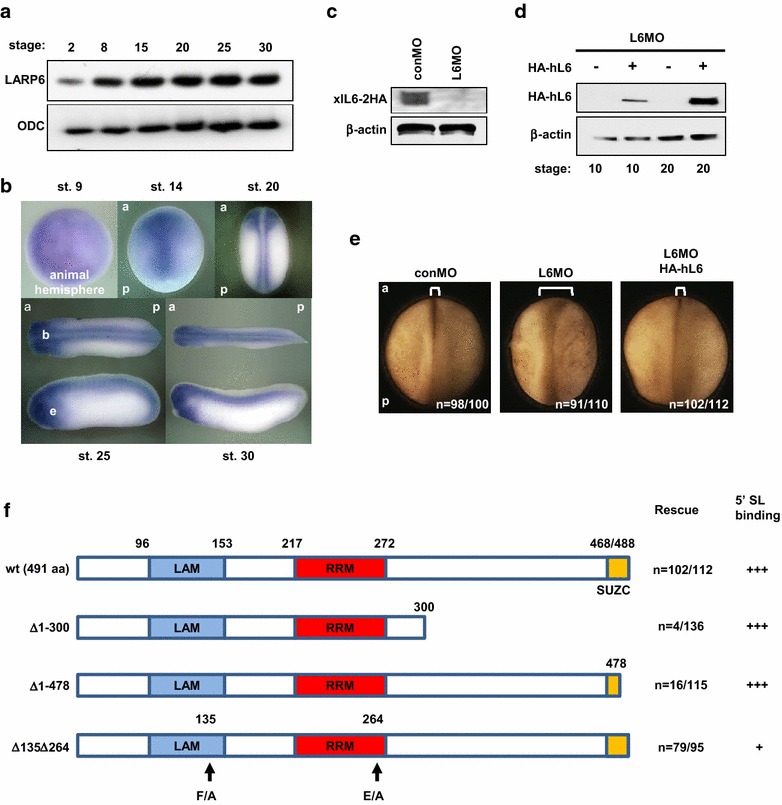
LARP6 morphants have neural tube closure defects. **a** Temporal expression profiles of *LARP6* gene. **b** Spatial expression profiles of *LARP6* gene. **c** LARP6 MO (L6MO) but not control MO (conMO) depleted *X. laevis* LARP6 protein (xlL6-2HA) in late neurula stage embryos. conMO or L6MO was injected with xlL6-2HA mRNA, and LARP6 was detected by immunoblotting with anti-HA antibody. **d** Human LARP6 (HA-hL6) was not knocked down by L6MO in stage 10 or 20 embryos. **e** Depletion of LARP6 resulted in neural tube closure defects. conMO, L6MO (15 ng/blastomere) and/or HA-hL6 mRNA (250 pg/blastomere) were injected into two dorsal blastomeres of 4-cell stage embryos (between animal pole and marginal zone). Images are dorsal views of late neurula stage embryos. **f** The summary of rescue experiments: the C-terminus of LARP6 is important for neural tube closure. *LAM* La motif, *RRM* RNA recognition motif, *SUZC* SUZ-C motif. *n* indicates number of embryos examined. *5′SL binding* shows the interaction between LARP6 and the 5′ stem-loop region of collagen RNA. *F* phenylalanine, *A* alanine, *E* glutamic acid, *a* anterior, *p* posterior, *b* brain, *e* eye. ODC and β-actin were used as a loading control

Currently, the best defined function of LARP6 in vertebrate is the translational regulation of type I collagen synthesis by binding to the 5′ stem-loop sequence of collagen mRNAs [12]. In *Xenopus* embryos, *LARP6* is maternally expressed and continues to be expressed throughout early *Xenopus* embryonic development (Fig. 1a). *LARP6* is strongly detected in the neural plate at the neurula stages as well as the central nervous system such as brain, spinal cord and eyes at the tailbud stages (Fig. 1b). Since type I collagen expression begins at the late neurula stage in *Xenopus* embryos [33], the expression pattern suggests that LARP6 may have additional functions during *Xenopus* development.

To assess the function of LARP6 in development, LARP6 protein was knocked down by injecting Morpholino Oligos against LARP6 (L6MO) (Fig. 1c). The effect of L6MO is highly specific to *Xenopus* LARP6, because it could not block the synthesis of the homologous human LARP6 protein (HA-hL6) expressed in *Xenopus* embryos (Fig. 1d). When L6MO was injected into the animal hemisphere of dorsal blastomeres at the 4-cell stage, the neural tube of LARP6 morphants failed to close (Fig. 1e). In addition, we could not test the role of LARP6 in myogenesis which was reported in moth, zebrafish and mice [11, 18], because the microinjection of LARP6 into the future dorsal mesoderm arrested their development by the tailbud stage. This neural tube closure defect of LARP6 morphants was rescued by co-injection of human LARP6, indicating that LARP6 is required for neural tube closure. Since the human LARP6 protein can restore this function, *Xenopus* LARP6 is its functional homolog. In addition to the La-module, LARP6 carries an SUZ-C motif (SUZC) [8, 9]. The SUZC in other RNA-binding protein is believed to contribute to mRNA substrate recognition [34]. To understand what domains are necessary for the function of LARP6 in neural tube closure, a rescue of the phenotype was performed with three different LARP6 mutants, Δ1-300, Δ1-478 and Δ135Δ264 (Fig. 1f). The interactions between these mutants and the 5′ stem-loop of type I collagen mRNA were previously assayed and summarized [12, 15, 21]. Following co-injection of L6MO and these mutant mRNAs, the neural tube formation of embryos was examined. Interestingly, Δ135Δ264 and wt but not Δ1-300 and Δ1-478 rescued neural tube closure defects in LARP6 morphants, indicating that the SUZC is required for the function of LARP6 in neural tube closure (Fig. 1f). Because this domain does not participate in binding collagen mRNAs and neural tube closure temporally precedes type I collagen expression, the molecular mechanism underlying this function is likely different from that for LARP6 in collagen synthesis.

### LARP6 morphants lack cilia at the neural tube

Neural differentiation, which forms the neural plate, is a fundamental embryonic process that leads to the development of the neural tube [35]. Therefore, we first examined neural differentiation in the neural plate. The expression of *sox2*, a pan-neural marker [36], was not changed in LARP6 morphants (Additional file 1: Figure S1a), indicating that LARP6 is not involved in neural differentiation at the neural plate. After the neural plate is formed, it lengthens along the anterior–posterior axis and narrows itself so the subsequent bending will form a tube. In *Xenopus* embryos, the convergent extension controls lengthening and narrowing of the neural plate [35]. Therefore, we tested if LARP6 controls the convergent extension by animal cap assay (Additional file 1: Figure S1b). The elongation of animal caps induced by activin was not blocked by knockdown of LARP6, suggesting that LARP6 does not control the convergent extension.

**Fig. 2:**
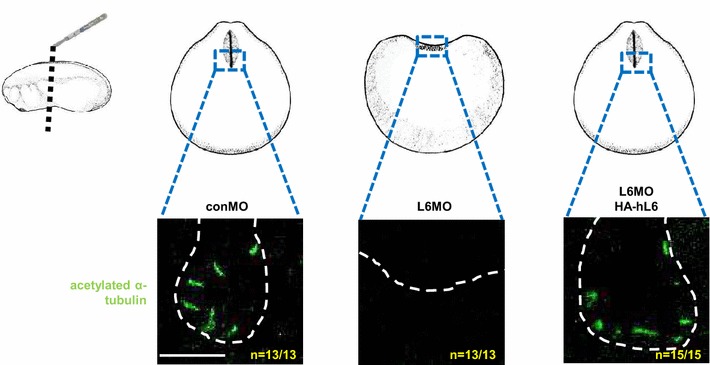
LARP6 is required for ciliogenesis in the neural tube. No cilia are detected in the neural tube of LARP6 morphants. conMO, L6MO and/or HA-hL6 mRNA were injected into two dorsal blastomeres of 4-cell stage embryos and fixed at stage 20. Cilia were stained by anti-acetylated α-tubulin antibody. Following staining, 100-μm transverse sections of specimens were prepared and images were taken by confocal microscopy. memRFP mRNA was injected as a lineage tracer. *White dots* indicate the lumen of the neural tube. The *scale bar* represents 10 μm. *n* indicates number of embryos examined

Recent studies have implicated cilia in the neural plate to be critical for the process of the neural tube closure [25, 37–39]. Therefore, cilia in the neural tube of LARP6 morphants were examined. After depletion of LARP6 by injection of L6MO, cilia were detected by immunostaining for acetylated α-tubulin [25]. Complete absence of cilia was observed in the neural tube of LARP6 morphants (middle column in Fig. 2). To show the specificity of this defect, an HA-hL6 mRNA was co-injected with L6MO to test for rescue. As we expected, cilia in the neural tube were restored upon supplementation with HA-hL6 expression (right column in Fig. 2). In addition to these ciliary morphologies, we also tested the gene expression of several cilia-related genes, such as *ARL13B*, *TTC25*, *Foxj1*, *RFX2*, *RFX4* and *RFX7* [25, 39–41]. The expression level of most cilia-related genes, with exception of *RFX2*, was reduced (Additional file 1: Figure S1c). These results indicated that neural tube closure defects in LARP6 morphants are coincident with disruption of neural tube ciliogenesis.

### LARP6 is required for the differentiation of MCCs

**Fig. 3:**
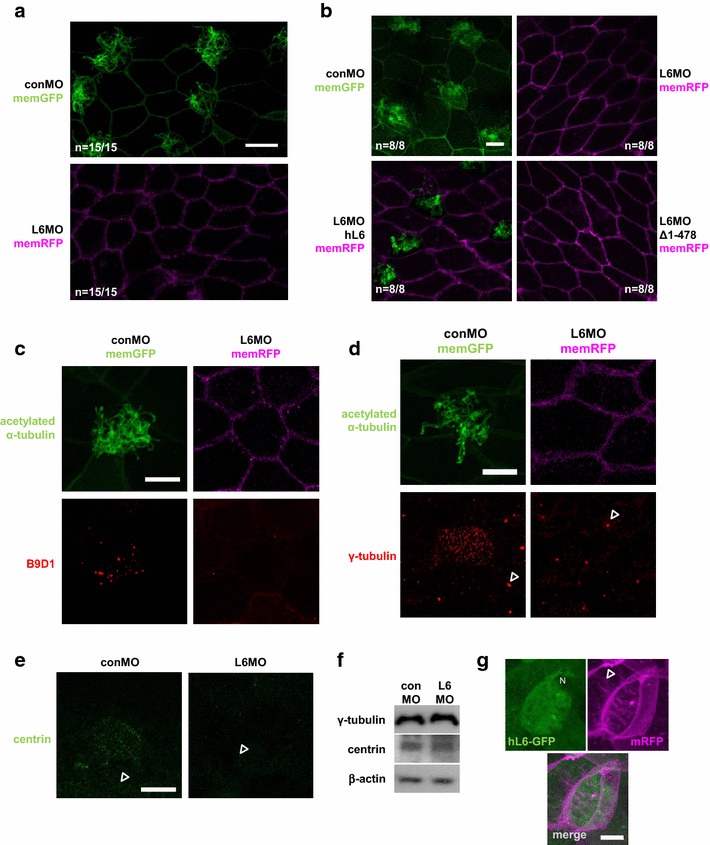
LARP6 is necessary for MCC differentiation. **a** Absence of cilia on epidermal MCC of LARP6 morphants. L6MO (15 ng/blastomere) with memRFP mRNA or conMO with memGFP mRNA were injected into the *right side* or the *left side*, respectively. Embryos were fixed at stage 25. Cilia were stained with an anti-acetylated tubulin antibody. Following staining, lateral explants were prepared from embryos and images from the *right* (L6MO) or *left* (conMO) lateral side were taken by confocal microscopy. **b** Rescue experiments with hL6 or Δ1-478 mRNA in LARP6 morphant MCCs. **c**–**e** B9D1, γ-tubulin and centrin proteins were not detected at the LARP6 morphant epidermis. Axonemes and ciliary transition zones or basal bodies were stained by an anti-acetylated tubulin and anti-B9D1, anti-γ-tubulin or anti-centrin antibody, respectively. Following staining, lateral explants were prepared from embryos and images were taken by confocal microscopy. *Arrowheads* indicate examples of centrioles. **f** The depletion of LARP6 did not change total amounts of γ-tubulin and centrin proteins in embryos. **g** The localization of LARP6 in MCCs. *N* nucleus. An *Arrowhead* indicates an example of a cilium. *Scale bars* 20 µm in (**a**), 10 µm in (**b**–**e**, **g**). *n* indicates number of embryos examined

Next, we tested whether the function of LARP6 was restricted to control ciliogenesis only in the neural tube or not. Here, we assessed the well-characterized cilia on the MCCs of the epidermis. When LARP6 was knocked down by L6MO, no cilia were observed at the epidermis (Fig. 3a), indicating that LARP6 is also involved in MCC ciliogenesis. To examine the specificity of L6MO effect in MCCs, rescue experiments with hL6 and Δ1-478 were performed (Fig. 3b). Co-injection of hL6 but not Δ1-478 mRNA restored MCCs in LARP6 morphant epidermis (Fig. 3b bottom panels), indicating that L6MO effect is specific to ciliary morphologies and the SUZC is also important for MCC differentiation. To further characterize the MCCs in LARP6 morphants, we examined the transition zone marker B9D1/MSKR1 [42–44] as well as the centriole/basal body markers γ-tubulin [45, 46] and centrin [47, 48] in LARP6 morphant MCCs. The transition zone and basal bodies are at the base of cilia, and serve as the ciliary gate or the ciliary base, respectively. Although γ-tubulin and centrin positive centrioles were detected at the LARP6 morphant epidermis (arrowheads in Fig. 3d, e), multiple signals of all three markers were not detected (Fig. 3c–e). In addition, the protein level of γ-tubulin and centrin was not changed in LARP6 morphants when analyzed by immunoblotting (Fig. 3f). These results suggest that LARP6 is required for the formation of basal bodies in the MCCs of the epidermis.

While LARP6 is expressed at the nucleus and the cytoplasm in mammalian cells [12], the localization of LARP6 in ciliated cells has not been reported previously. To examine the localization of LARP6 in MCCs, we expressed GFP-fused human LARP6 (hL6-GFP) with mRFP, which can label cell membranes and cilia [49], in epidermis of *Xenopus* embryos. LARP6 was dominantly detected in the cytoplasm but not cilia of MCCs (Fig. 3g), suggesting that LARP6 may control translation of ciliary or cilia-related factors in MCC differentiation.

### *mcidas* is a downstream factor of LARP6

**Fig. 4:**
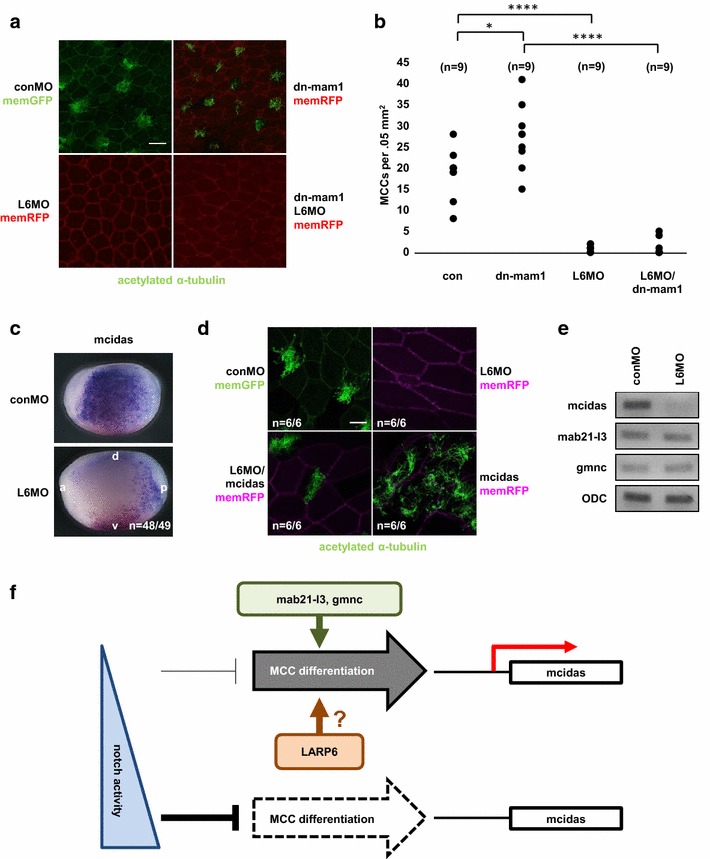
LARP6 regulates the expression of *mcidas* in ciliogenesis. **a** The depletion of LARP6 blocked ciliogenesis in a Notch-independent manner. dn-mam1 RNA and/or L6MO with memRFP mRNA or conMO with memGFP mRNA were injected into the *right side* or the *left side*, respectively. Embryos were fixed at stage 25. Cilia were stained with an anti-acetylated tubulin antibody. Following staining, lateral explants were prepared from embryos and images were taken by confocal microscopy. The *scale bar* represents 20 μm. **b** Quantification of number of MCCs from **a**. Three embryos were randomly chosen from each of three independent experiments. *≤0.05, ****≤0.0001. **c** *mcidas* expression in LARP6 morphants at stage 14 by WISH. *Images from the left* (conMO) or *right* (L6MO) lateral side were taken from the same embryo. Red-gal signals indicate L6MO injected side. *a* anterior, *p* posterior, *d* dorsal, *v* ventral. **d** Rescue experiments with mcidas mRNA (500 pg/blastomere) in LARP6 morphant MCCs. **e** LARP6 does not control the expression of *mab21*-*l3* and *gmnc* genes. L6MO was injected into two blastomeres of 2-cell stage embryos and RNAs were isolated at stage 13 for semi-quantitative RT-PCR. **f** The model of LARP6 function in ciliogenesis. LARP6 controls the expression of *mcidas* in a Notch-independent manner. *n* indicates number of embryos examined

To define the role of LARP6 within the established molecular network of MCC ciliogenesis, we first examined the connection to the Notch signaling pathway. Notch signaling has been previously shown to restrict the formation of MCCs at the epidermis [50] and suppress the expression of the transcription regulator *mcidas* which initiates MCC differentiation [51, 52]. When Notch signaling is inhibited by a dominant negative form of the co-transcriptional activator mastermind 1 (dn-mam1) [53], the density of MCCs slightly increased (~2 times) [50, 54]. To test if LARP6 controls MCC differentiation in a Notch-dependent manner, we examined the effect of LARP6 depletion on ciliogenesis at the epidermis when Notch signaling was blocked by dn-mam1. Expression of dn-mam1 alone slightly increased the number of MCCs, but co-injection of dn-mam1 and L6MO completely blocked the formation of MCCs (Fig. 4a, b). If LARP6 blocks Notch signaling to promote MCC differentiation, we expected that increased number of MCCs by injection of dn-mam1 would not be changed. However, co-injection of L6MO resulted in absence of cilia, indicating that LARP6 controls MCC differentiation downstream of Notch signaling, or in a Notch-independent manner. Furthermore, the expression of an active form of Notch receptor, the Notch receptor intracellular domain (NICD), did not suppress the expression of *LARP6*, in contrast to what was observed for *mcidas* (*multicilin*) [51] (Additional file 1: Figure S1d), suggesting that the expression of *LAPR6* is not regulated by Notch signaling. Taken together, these results implicate that LARP6 is likely to control MCC differentiation in a Notch-independent manner rather than downstream of Notch signaling.

We next examined whether LARP6 controls the expression of *mcidas* in MCCs, as mcidas is a key factor that promotes MCC differentiation. The expression of *mcidas* was inhibited in LARP6 morphants (Fig. 4c), further suggesting that LARP6 is required for MCC differentiation at the early stage of this process. In addition, we did not observe an interaction between LARP6 and *mcidas* mRNA (data not shown). To test if mcidas mediates the function of LARP6 in MCC differentiation, rescue experiments with mcidas in LARP6 morphant MCCs were performed (Fig. 4d). Co-injection of *mcidas* mRNA restored MCCs in LARP6 morphant epidermis, but the number of MCCs was less than in mcidas-expressed epidermis (Fig. 4d, bottom panels). This result indicates that mcidas is functionally downstream of LARP6 but additional downstream factors of LARP6 are required for MCC differentiation. In two recent studies, mab21 family gene mab21-like3 (mab21-l3) and geminin coiled-coil domain containing (gmnc) have been shown to control the expression of *mcidas* and regulate MCC ciliogenesis [55, 56]. Therefore, we assessed if LARP6 has a connection with these molecules to control the expression of *mcidas*. The expression of *mab21*-*l3* and *gmnc* genes was not changed in LARP6 morphants (Fig. 4e) and the interaction between LARP6 and mRNAs of *mab21*-*l3* or *gmnc* was not detected by immunoprecipitation (data not shown), indicating that the regulation of *mcidas* gene expression by LARP6 is not mediated by *mab21*-*l3* and *gmnc* expression.

Taken together, our results show that LARP6 controls the early stage of MCC differentiation through a mechanism that appears to be independent of Notch signaling (Fig. 4f) and mcidas is an important downstream factor of LARP6 in this process.

## Discussion

The results presented here reveal a role of La-related protein 6 (LARP6) for ciliated cell differentiation in *Xenopus* embryos. Knockdown of LARP6 resulted in neural tube closure defect, which is coincident with the absence of cilia, and blocking MCC differentiation at the epidermis. Although LARP6 appears to control MCC differentiation through a yet-discovered molecular network, mcidas is an important downstream factor of LARP6 in this process. Since the molecular mechanisms underlying the structure and function of cilia are well-conserved among the vertebrates [3], this newly discovered role of LARP6 in ciliated cell differentiation is likely conserved in humans and may be involved in pathogenesis of human ciliopathies.

Knockdown of LARP6 in *Xenopus* embryos resulted in neural tube closure defects. The first step of neural tube closure is neural differentiation in dorsal ectoderm of embryos which becomes the neural plate. Neural differentiation, which was represented by the expression of sox2, was not changed in LARP6 morphants. After neural differentiation, the neural plate buckles, rolls up and then fuses to form a hollow tube called the neural tube [35]. The convergent extension is involved in shaping of the neural plate of *Xenopus* embryos which is required for the formation of a tube. Our study indicated that LARP6 was not required for the convergent extension. These results suggested that neural tube closure defects in LARP6 morphants are caused by neither the inhibition of neural differentiation in dorsal ectoderm nor shaping of the neural plate. Therefore, neural tube closure defects of LARP6 morphants may be caused by the loss of cilia.

LARP6 has the LAM and the RRM, collectively termed the La-module, which is required for binding to the 5′ stem-loop of type I collagen mRNAs [12], as well as the SUZC in the extreme C-terminus. Although the SUZC is not necessary for binding the 5′ step-loop of collagen mRNAs, this motif was required for neural tube closure and MCC differentiation. Moreover, LARP6 mutant Δ135Δ264, which weakly interacts with the 5′ stem-loop of collagen I mRNAs [15], could rescue neural tube closure defect of LARP6 morphants similar to wild-type LARP6. These data indicate that LARP6 may regulate mRNAs for ciliated cell differentiation through a different mechanism than the mechanism which regulates collagen synthesis. However, at present we have not yet identified the target mRNAs for LARP6 required for ciliated cell differentiation. The identification of LARP6 target mRNAs in development will be critical to uncover the molecular mechanism underlying ciliated cell differentiation controlled by LARP6.

The SUZC of LARP6 is required for the differentiation of ciliated cells at the neural tube and MCCs. Since this motif in other protein is generally believed to contribute to mRNA substrate recognition [34], factor(s) interacting with the SUZC of LARP6 must be important for the differentiation of ciliated cells and may determine the target mRNA(s) in this process. Serine–threonine kinase receptor-associated protein (STRAP) has been previously shown to interact with the SUZC of LARP6 [16]. STRAP was initially identified as a novel WD40 domain-containing protein which interacts with both TβR-I and TβR-II serine–threonine kinase receptors and negatively regulates gene expression from TGF-β-responsive promoters [57]. Although homozygous STRAP mutant embryos are embryonically lethal with many defects between embryonic day 10.5 and 12.5 [58], these embryos present neural tube closure defects. Therefore, STRAP seems to be a possible partner of LARP6 in the differentiation of ciliated cells. However, further studies are required to reveal the importance of this SUZC and identify interacting factor(s) during the differentiation of ciliated cells.

mcidas has been reported to control centriole assembly and promote MCC differentiation [51, 52]. MCCs with multiple basal bodies were lost in the epidermis of LARP6 morphants, and mcidas appears to mediate this function of LARP6 partially. Although mcidas could restore MCCs in LARP6 morphants, the number of MCCs induced by mcidas was reduced in LARP6 morphants, indicating that additional factor(s) may be needed for complete restoration of MCCs in LARP6 morphants. Therefore, LARP6 appears to control the expression of *mcidas* and *additional factor(s)* which cooperate with mcidas in MCC differentiation. In addition, mcidas is not expressed in the neural tube [51], but cilia were absent in LARP6 morphant neural tube. This also indicates that LARP6 controls the expression of additional factor(s) which are necessary for the differentiation of ciliated cells. For instance, our study showed that the expression of *RFX4* and *7*, which are required for neural tube ciliogenesis [25], is controlled by LARP6. Furthermore, while the expression of *mcidas* and MCC differentiation are negatively regulated by Notch signaling [51], the increase of MCC formation induced by inhibition of Notch signaling was completely blocked by knockdown of LARP6. These results indicate that LARP6 likely controls the expression of *mcidas* and MCC differentiation in a Notch-independent manner. mab21-l3 and gmnc proteins are known to regulate the expression of *mcidas* and MCC differentiation [55, 56], however, we did not observe any changes in the expression level of these genes in LARP6 morphants and the interaction between LARP6 and their mRNAs. Therefore, LARP6 is likely involved in *mcidas* expression and MCC differentiation in a yet-uncovered mechanism (Fig. 4f). The identification of LARP6 target mRNAs will help us to understand the molecular mechanism of ciliated cell differentiation.

## Conclusions

In summary, the work presented here strongly suggests that LARP6 is involved in the differentiation of ciliated cells and that this function is independent of the regulation of type I collagen synthesis. Moreover, recent single-nucleotide polymorphisms’ analysis from four genome-wide association studies indicate that a polymorphism at or near LARP6 is linked to the risk of type 2 diabetes [59]. Since Bardet–Biedl syndrome, a ciliopathic human genetic disorder, has been reported to be linked to obesity and diabetes [60], dysfunction of LARP6 may result in ciliopathies associated with type II diabetes. In this aspect, our study opens new avenues to investigate the pathogenesis of ciliopathies.

## Additional file

**Additional file 1: Figure S1.** LARP6 controls the expression of cilia-related genes in a Notch-independent manner.

## Abbreviations

LARP6: La-related protein 6
RBP: RNA-binding protein
BisC: Bicaudal C
LAM: La motif
RRM: RNA recognition motif
STRAP: serine–threonine kinase receptor-associated protein
FKBP3: FK506 binding protein 3
MCC: multi-ciliated cell
SUZC: SUZ-C motif
NICD: Notch receptor intracellular domain
mab21-l3: mab21 family gene mab21-like3
gmnc: geminin coiled-coil domain containing
