# Supplementary material for: La-related protein 6 controls ciliated cell differentiation
Source: Cilia. 2017 Mar 23;6:4. doi: 10.1186/s13630-017-0047-7 (PMC5364628; doi:10.1186/s13630-017-0047-7)
Supplement: Supplementary file 1 — Additional file 1: Figure S1. LARP6 controls the expression of cilia-related genes in a Notch-independent manner. [file 13630_2017_47_MOESM1_ESM.pdf]

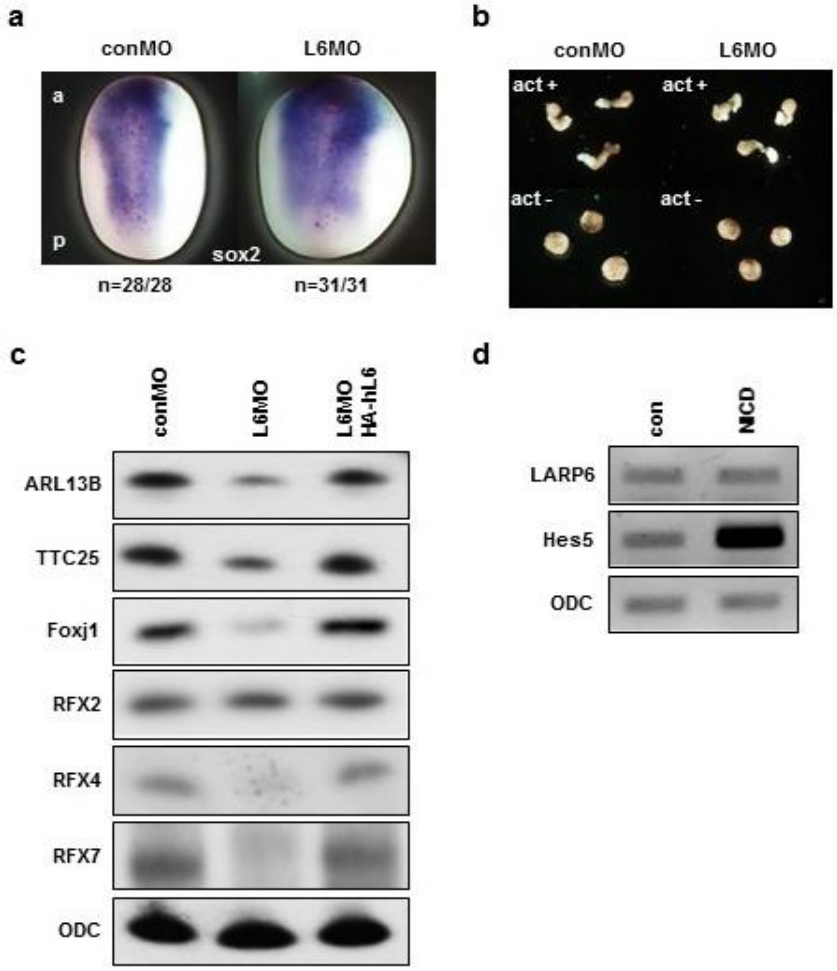

Figure S1. LARP6 controls the expression of cilia-related genes in a Notch-independent manner. (a) LARP6 is not necessary for the expression of *sox2*, a pan-neural marker [1], in neurula stage embryos. Images are dorsal views. a: anterior, p: posterior. (b) LARP6 does not control the convergent extension in animal caps. The elongation of caps induced by activin (50 ng/ml) was not blocked by depletion of LARP6. Animal cap assay was performed as described in previous work [2]. (c) The expression of ciliary genes and key transcription factors in LARP6 morphants. conMO, L6MO and/or HA-hL6 mRNA were injected into two blastomeres of two-cell stage embryos and total RNAs were isolated at the late neurula stage. RNAs were used for semi-quantitative RT-PCR. (b) Notch signaling does not control the expression of *LARP6* gene. NICD mRNA (100 pg) was injected into two blastomeres of two-cell stage embryos and RNAs were isolated at stage 13. RNAs were used for semi-quantitative RT-PCR. *Hes5* expression shows the activation of Notch signaling. Sequences of primers used; Foxj1: Forward 5’-CTCCCAAGAGGAGCAAACTG-3’, Reverse 5’- CTTTCCATGAACGGTGAGGT-3’;

RFX2: Forward 5'- TGCTTCAATGTCTCCTGTGC-3', Reverse 5'-AGGCAGACTGCATCAGAGGT-3'. Other primers were used in previous publication [3, 4]

References

1. Mizuseki K, Kishi M, Matsui M, Nakanishi S, Sasai Y: **Xenopus Zic-related-1 and Sox-2, two factors induced by chordin, have distinct activities in the initiation of neural induction**. *Development (Cambridge, England)* 1998, **125**(4):579-587.
2. Kato Y, Shi Y, He X: **Neuralization of the Xenopus embryo by inhibition of p300/ CREB-binding protein function**. *J Neurosci* 1999, **19**(21):9364-9373.
3. Manojlovic Z, Earwood R, Kato A, Stefanovic B, Kato Y: **RFX7 is required for the formation of cilia in the neural tube**. *Mechanisms of development* 2014, **132**:28-37.
4. Tozser J, Earwood R, Kato A, Brown J, Tanaka K, Didier R, Megraw TL, Blum M, Kato Y: **TGF-beta Signaling Regulates the Differentiation of Motile Cilia**. *Cell reports* 2015, **11**(7):1000-1007.
